# Thermo-Magnetostrictive Effect for Driving Antiferromagnetic Two-Dimensional Material Resonators

**DOI:** 10.1021/acs.nanolett.3c01610

**Published:** 2023-07-19

**Authors:** Gabriele Baglioni, Makars Šiškins, Maurits Houmes, Martin Lee, Dong Hoon Shin, Samuel Mañas-Valero, Eugenio Coronado, Yaroslav M. Blanter, Herre S. J. van der Zant, Peter G. Steeneken

**Affiliations:** † Kavli Institute of Nanoscience, Delft University of Technology, Lorentzweg 1, 2628 CJ Delft, The Netherlands; ‡ Instituto de Ciencia Molecular (ICMol), Universitat de Valencia, Catedrático José Beltrán 2, 46980 Paterna, Spain; § Department of Precision and Microsystems Engineering, Delft University of Technology, Mekelweg 2, 2628 CD Delft, The Netherlands

**Keywords:** nanomechanics, magnetic materials, phase transitions, two-dimensional materials

## Abstract

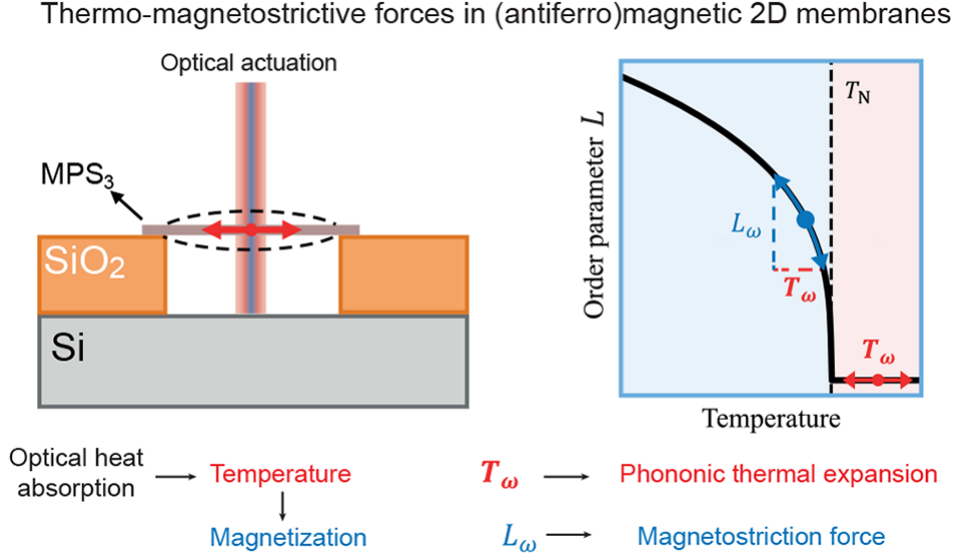

Magnetostrictive coupling has recently attracted interest as a sensitive method for studying magnetism in two-dimensional (2D) materials by mechanical means. However, its application in high-frequency magnetic actuators and transducers requires rapid modulation of the magnetic order, which is difficult to achieve with external magnets, especially when dealing with antiferromagnets. Here, we optothermally modulate the magnetization in antiferromagnetic 2D material membranes of metal phosphor trisulfides (MPS_3_), to induce a large high-frequency magnetostrictive driving force. From the analysis of the temperature-dependent resonance amplitude, we provide evidence that the force is due to a thermo-magnetostrictive effect, which significantly increases near the Neél temperature, due to the strong temperature dependence of the magnetization. By studying its angle dependence, we find the effect is observed to follow anisotropic magnetostriction of the crystal lattice. The results show that the thermo-magnetostrictive effect results in a strongly enhanced thermal expansion force near the critical temperature of magnetostrictive 2D materials, which can enable more efficient actuation of nano-magnetomechanical devices and can also provide a route for studying the high-frequency coupling among magnetic, mechanical, and thermodynamic degrees of freedom down to the 2D limit.

Given the strong interest in applications of magnetic devices for sensing, data storage, and spintronics, two-dimensional (2D) magnetic materials have been the subject of extensive research.^1^ However, testing the magnetic properties of suspended 2D materials is challenging due to the small sample volumes, which result in small signals. As an alternative to optical and electronic techniques, recently several works have promoted the use of nanomechanical methods for probing the magnetic and thermodynamic properties of suspended 2D membrane resonators such as graphene,^2^ MoS_2_,^3^ and MoSe_2_.^4^ Owing to the strong magnetostrictive coupling between mechanical strain and magnetism in certain 2D materials,^5,6^ nanomechanics has also been used to probe the magnetic properties^5−8^ and anisotropies^9^ via resonance frequency measurements.

Instead of probing magnetism by mechanical resonance, in this work, we demonstrate the inverse mechanism: driving mechanical resonators via 2D magnetic order through magnetostriction. Magnetostriction is the expansion of a material as a result of a change in its magnetization. It is expected that when a sufficiently large modulation of the magnetization in a 2D material resonator is generated, this will lead to a force that can drive it into resonance. However, generating a sufficiently large magnetization with an external magnet is not trivial, both because of the relatively high resonance frequencies (>10 MHz) of ultrathin membranes and because the antiferromagnetic nature of the 2D materials under study makes them insensitive to external fields.

Therefore, we use temperature modulation as a means to change the magnetization and drive the resonators by a thermo-magnetostrictive effect. This is especially efficient just below the Neél temperature of the antiferromagnetic MPS_3_ (M = Fe, Co, or Ni) resonators under study, where the staggered magnetization is strongly dependent om temperature. We show that by optothermal modulation of the temperature of the suspended 2D membranes, the magnetization can be varied at high frequencies, causing a large magnetostrictive enhancement of the driving force, which follows the anisotropy of the magnetostriction coefficient.

We study the mechanical motion of suspended MPS_3_ membranes by driving them with a power-modulated blue laser while detecting their motion interferometrically with a red laser.^2,3^ Three different types of antiferromagnetic 2D materials are studied: circular drum resonators of FePS_3_ and NiPS_3_ and rectangular membranes of CoPS_3_ arranged in a star-shaped array for studying the effect of anisotropy. See Supporting Information A for more details on the experimental setup (Figure S1) and measured samples (Table S1).

Panels a and b of Figure 1 show an optical image of a FePS_3_ circular drum (sample Fe-1 in Table S1) and a schematic cross section of the device under study. MPS_3_ flakes are stamped over a cavity in a prepatterned Si/SiO_2_ substrate and are optothermally actuated by an optical power *P*_ω_ = *P*_0_e^*iωt*^ that is modulated at angular frequency ω. A typical resonance peak of the actuated membrane is shown in Figure 1c, along with the damped harmonic oscillator fit used to extract its resonance frequency, ω_0_/2π, and *Q* factor.

**Figure 1:**
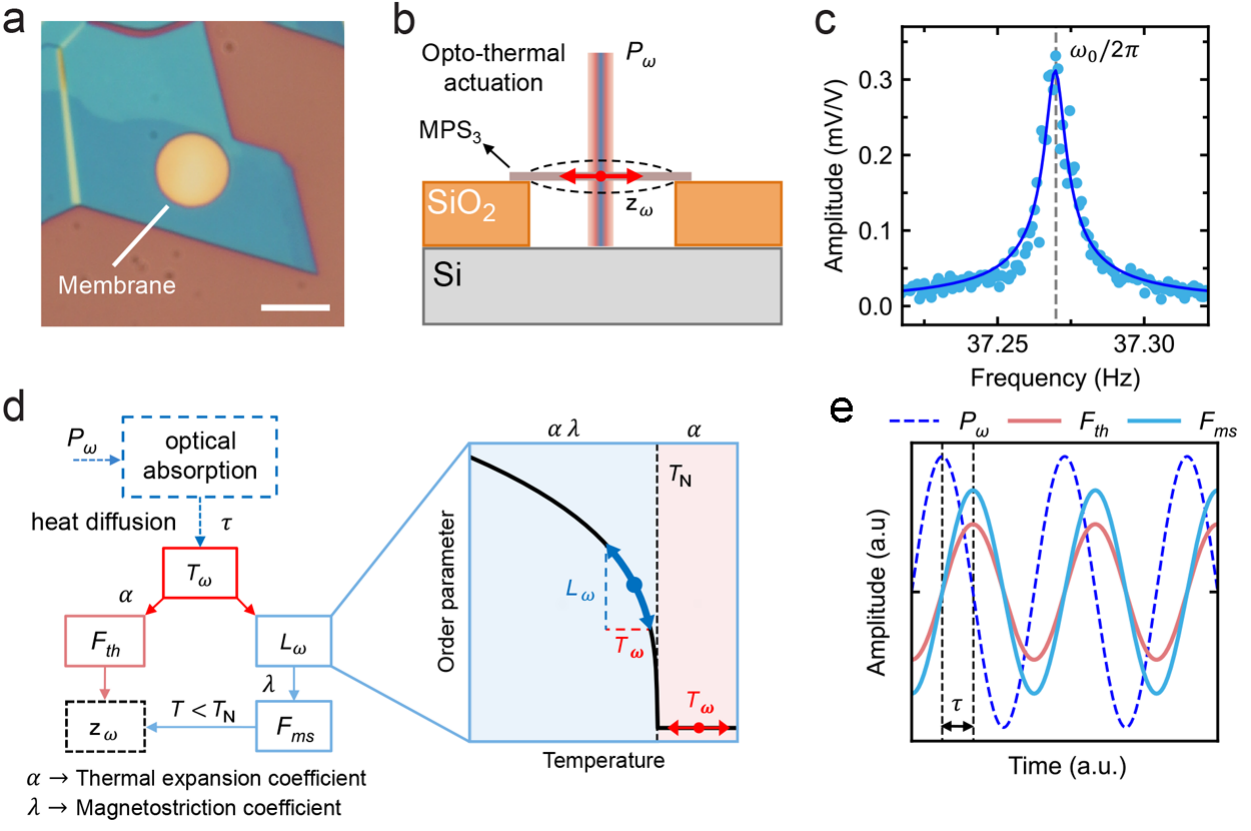
Thermal response characterization of magnetic membranes. (a) Optical image of a FePS_3_ membrane resonator (sample Fe-1 in Table S1). The scale bar is 6 μm. (b) Schematic illustration of the system under study. The MPS_3_ membrane is optothermally actuated via a power-modulated blue laser, *P*_ω_, and its out-of-plane displacement, *z*_ω_, is detected interferometrically with a red laser. The red arrows show the expansion direction. (c) Measured amplitude of the fundamental resonance peak (at 120 K) and fit (drawn line) used to extract the resonance frequency, ω_0_/2π, whose position is indicated by the vertical dashed line. (d) Block diagram showing how thermal expansion forces, *F*_th_, and magnetostriction forces, *F*_ms_, arise from the optothermal actuation and contribute to the membrane’s motion. *T*_ω_ and *L*_ω_ indicate the modulation of the temperature and magnetic order parameter, respectively. (e) Schematic plot of the blue laser power, *P*_ω_, and out-of-phase forces, *F*_th_ and *F*_ms_, resulting from time delay τ due to heat diffusion.

As schematically shown in panels d and e of Figure 1, the forces actuating the membrane arise from delayed temperature modulation, caused by the absorption of the blue laser power. The delayed actuation results in a thermal peak^2^ in the imaginary part of the mechanical displacement spectrum *z*_ω_ at ω = 1/τ ≪ ω_0_ described by , where amplitude *A* is proportional to the driving force while τ is the membrane’s thermal time constant, which is proportional to the material’s thermal diffusivity.^10,11^ Here, we assume that time delay τ between optical power *P*_ω_ and membrane displacement *z*_ω_ is dominated by thermal effects.^2^ Other sources of delay are analyzed in Supporting Information B. The complete actuation mechanism is discussed in more detail below.

The frequency-dependent motion of the membrane, *z*_ω_, is measured by a red laser with interferometric readout, via a diode detector voltage *V*_out_ and network analyzer that determines the light intensity modulation (Figure S1). The power modulation voltage, *V*_in_, of the blue laser that drives the membrane optothermally is kept at a constant amplitude. We investigate the frequency and amplitude response of the resonator over a wide frequency range to resolve both the device resonance frequency and its thermal peak below resonance.

Figure 2a shows the real and imaginary parts of the response spectra *V*_out_/*V*_in_ ∝ *z*_ω_ at different temperatures, for the FePS_3_ sample Fe-1 shown in Figure 1a. The data are fitted^2^ by the theoretical function  (in black). The imaginary part of *z*_ω_ exhibits a peak with an amplitude of *A* at frequency ω_th_/(2π) = 1/(2*πτ*). Fitting data at every temperature, similar to those in Figure 2a, yields τ(*T*) and *A*(*T*), as shown in panels b and c, respectively, of Figure 2 for sample Fe-1. Interestingly, the thermal time constant displays a maximum at the Neél temperature *T*_N_ of FePS_3_ indicated by the dashed line. Just below *T*_N_, amplitude *A* of the resonator shows a large peak, which is larger by a factor of >20 than its value at 70 K. In the following sections, we will first analyze thermal time constant τ(*T*) and its peak near *T*_N_ and subsequently the large peak in *A*(*T*).

**Figure 2:**
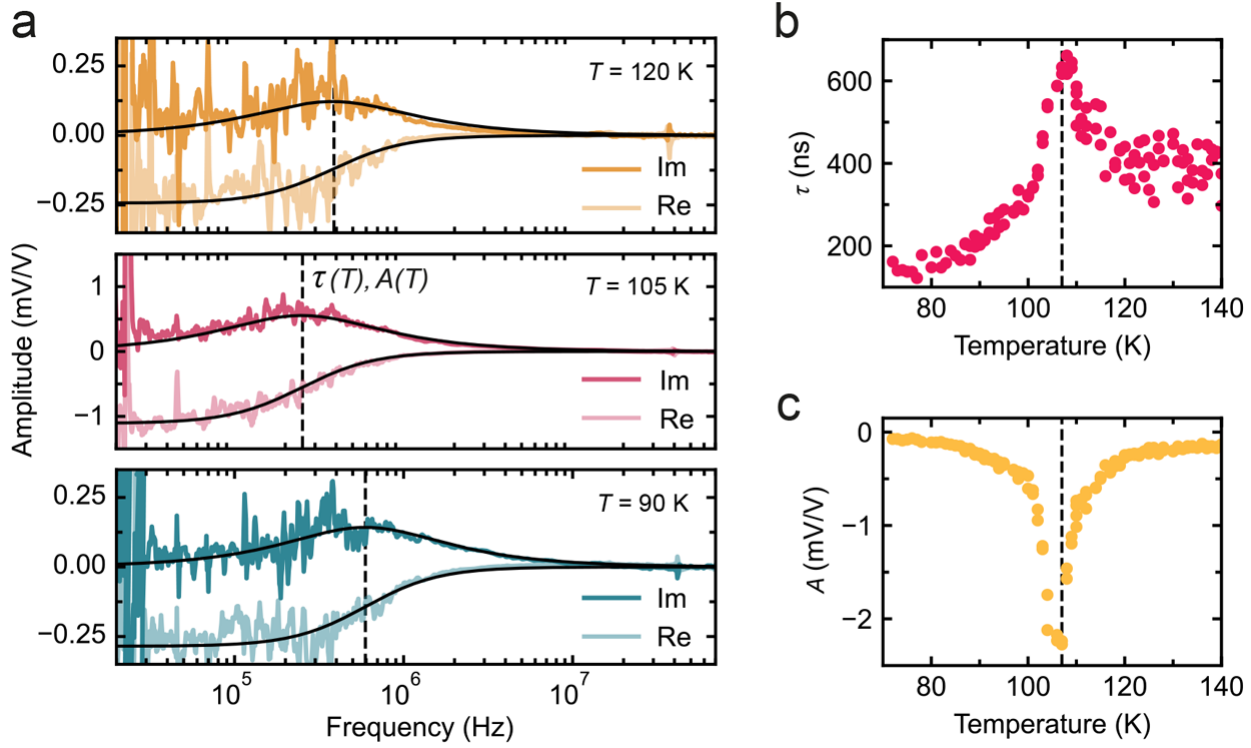
Thermal response vs temperature. Measurement data and analysis from FePS_3_ sample Fe-1. (a) Measured real and imaginary component of *z*_ω_ as a function of frequency at different temperatures (90 K for the bottom panel, 105 K for the middle panel, and 120 K for the top panel, fit parameters of τ = 267, 634, and 420 ns and *A* = −0.28,–1.11, and −0.24 mV/V, respectively). The black solid line indicates the fit to *A*/(1 + *iωτ*), which is used to extract τ and *A*. Vertical dashed lines highlight the frequency position of the thermal peak, ω_th_/(2π) = 1/(2*πτ*). (b and c) Thermal time constant τ and thermal peak amplitude *A*, respectively, as a function of temperature, extracted from data like those in panel a. Vertical dashed lines indicate the transition temperature, *T*_N_.

Given that the specific heat of FePS_3_ shows a peak near the antiferromagnetic phase transition,^6^ whether the anomaly in the specific heat can account for the peak in the thermal time constant in Figure 2b is of interest. As experimentally verified,^3^ the thermal time constant of a circular membrane is approximately given by
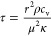
1where *r* is the membrane’s radius, *c*_v_, ρ, and κ are the material’s specific heat, density, and thermal conductivity, respectively, and μ^2^ is a constant related to the membrane geometry. Figure 3a shows the literature values^12^ of thermal conductivity κ(*T*) of bulk FePS_3_ and the material’s specific heat  calculated from the measured resonance frequency, ω_0_/2π, according to the methodology outlined in ref (6) for sample Fe-1 (see also Supporting Information C). In Figure 3b, we use eq 1 with *r* = 3 μm, ρ = 3375 kg m^–3^, and μ^2^ = 10 and the values of κ and *c*_v_ in Figure 3a to calculate thermal time constant τ (black curve) and compare it to the measured values (red dots). Equation 1 yields a good correspondence with the experimental data, reproducing the peak shape, as well as its magnitude. The qualitative correspondence between the modeled and measured data for τ(*T*) in Figure 3b provides evidence that the measured peak in the thermal time constant is due to the peak in the specific heat near *T*_N_. Further analysis of the thermal properties of the membranes can be found in Figures S3 and S4.

**Figure 3:**
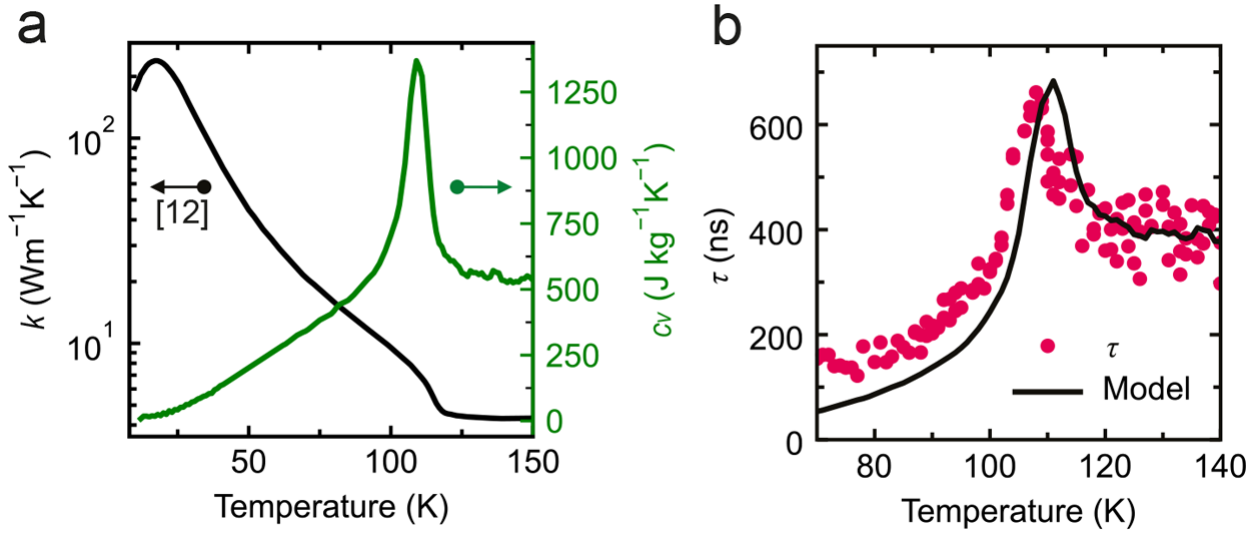
Thermal time constant model. Measurement data and analysis from FePS_3_ sample Fe-1. (a) Bulk thermal conductivity κ (black) from ref (12) (data adapted with permission) and measured specific heat *c*_v_ (green) extracted from the resonance frequency, ω_0_/2π(*T*), as described in ref (6) for FePS_3_. (b) Thermal time constant for the same sample as in panel a, calculated from eq 1 (black line), with μ^2^ = 10, *c*_v_, and κ from panel a and measured (red dots).

After having discussed temperature-dependent thermal time constant τ(*T*) from Figure 2b, we now turn to the large enhancement of the resonator’s amplitude near *T*_N_ observed in Figure 2c. To understand this enhancement, we first note that peak height *A*(*T*) is determined at a frequency ω_th_ = 1/τ that is far below the resonance frequency. At this frequency, the effects of mass and damping can be neglected, and the resonator behaves as a spring with stiffness *k*, and displacement *z*_ω,th_ = *F*_ω,th_/*k* ∝ *A*. Because ω_0_^2^ ∝ *k* and there is no peak in the resonance frequency near *T*_N_ (see Figure S2a in Supporting Information C), we conclude that the peak in *A*(*T*) should be attributed to a large enhancement in the driving actuation force near *T*_N_.

The proposed actuation mechanism is schematically depicted in panels d and e of Figure 1. The absorption of the power-modulated laser light supplies time-dependent heating power to the membrane. As a consequence, heat flows from the center of the membrane to the substrate on a time scale determined by the materials’ thermal time constant τ. Normally, the resulting temperature rise, *T*_ω_, increases the lattice vibrations that result in an enlarged lattice constant proportionally to effective lattice thermal expansion coefficient α of the membrane. However, in the case of an antiferromagnetic membrane below the Néel temperature, *T*_ω_ also changes the (staggered) magnetization order parameter, *L*. If the material is magnetostrictive, then its lattice expands in proportion to *λL*^2^, where λ is the effective magnetostriction coefficient of the membrane.

Thus, when the temperature is optothermally modulated below *T*_N_, the membrane is actuated both by the lattice thermal expansion force, *F*_th_, and by the magnetostrictive force, *F*_ms_, which are both delayed by characteristic time constant τ with respect to heating power *P*_ω_, as shown in Figure 1e. Because the slope of the magnetization versus temperature curve, *L*(*T*), is steepest just below *T*_N_, it is expected that the contribution of *F*_ms_ will also be largest in this temperature range, consistent with the peak in *A*(*T*) observed in Figure 2c.

To analyze the data and models in more quantitative detail, we derive the following equation for the low-frequency (ω ≪ ω_0_) mechanical displacement spectrum, *z*_ω_, in Supporting Information E:
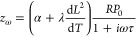
2where *R* is the membrane’s thermal resistance. As eq 2 shows, the thermal peak amplitude *A*(*T*) = Im(*z*_ω_) at ω = 1/τ is determined by two terms: one containing effective thermal expansion coefficient α and the other containing the temperature derivative of magnetic order parameter *L*^2^. Coefficient α can be expressed in terms of the specific heat considering the thermodynamical relation , where γ is the Grüneisen parameter, *K* the bulk modulus, and *V*_M_ the molar volume. Assuming that the isotropic contributions to the thermal expansion causing the membrane’s motion are determined by only the phononic lattice contribution, then α ∝ *c*_debye_, where *c*_debye_ is the debye specific heat:
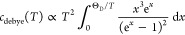
3For the analysis of the FePS_3_ data, we use a debye temperature Θ_D_ of 236 K,^13^ while for CoPS_3_, we use a Θ_D_ of 262 K as estimated from the material bulk modulus.^14^

To fit the data using eq 2, we employ the method from ref (9) to extract *L*^2^ from the angle-resolved resonance frequency of rectangular resonators (see also Supporting Information F for more details of this analysis). Figure 4a shows the fits of the *A*(*T*) data of a rectangular CoPS_3_ resonator of sample Co-1, which is shown in Figure 4c, to  with *a*_1_ and *a*_2_ as fit parameters. The dashed lines highlight the individual contributions to *A* from the thermal expansion (light red) and magnetostriction forces (light blue). The fitted curve (black) corresponds well to the measured *A*(*T*) data, providing evidence of the correctness of eq 2 and the applicability of the presented analysis. Figure 4b shows the same analysis on *A*(*T*) for FePS_3_ sample Fe-1, again resulting in good correspondence to the data. In this case, because *A*(*T*) is measured on a circular FePS_3_ drum on which *L*(*T*) is difficult to obtain using the method from ref (9), *L*^2^ is calculated from resonance frequency measurements on separate rectangular FePS_3_ resonators (as shown in Figure S8 of Supporting Information F), assuming the same temperature dependence of the magnetization holds for the circular resonator. Supporting Information G shows additional data on all rectangular cavities of sample Co-1 (Figure S9), as well as another FePS_3_ drum (sample Fe-2 in Figure S10) and NiPS_3_ drums (samples Ni-1–3 in Figure S11), demonstrating the reproducibility of the effect.

**Figure 4:**
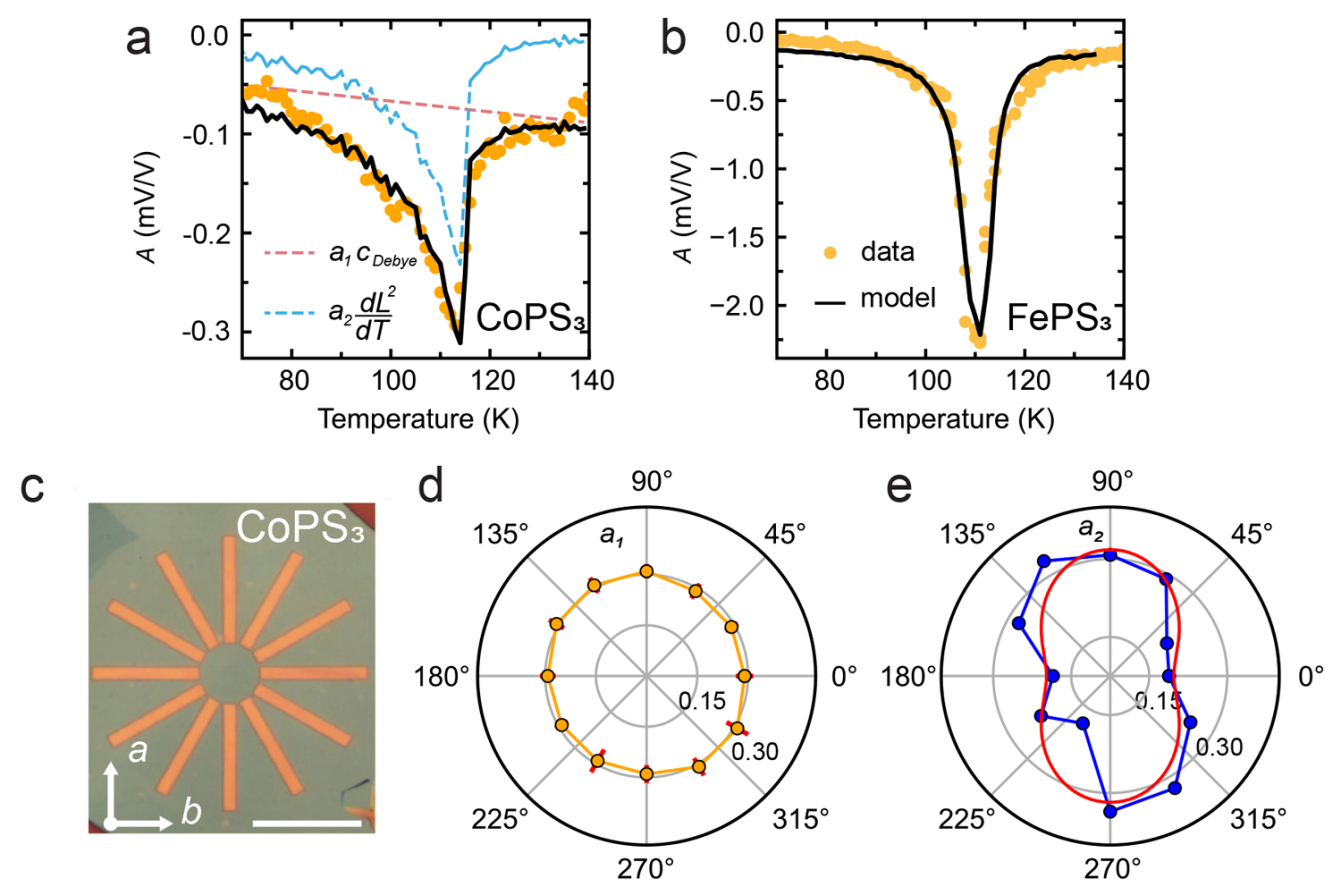
Actuation mediated by the magnetostriction effect. (a) Measured thermal peak amplitude *A* (orange) with a fit to  (black) on a rectangular resonator of CoPS_3_ sample Co-1, where *a*_1_ and *a*_2_ are fit parameters. The dashed lines indicate the individual contributions of the debye term (light red) and magnetostriction term (light blue). (b) Same as panel a for FePS_3_ sample Fe-1. (c) Optical image of the star-shaped array resonators of CoPS_3_ (sample Co-1) measured to investigate the angle dependence of *a*_1_(θ) and *a*_2_(θ). The scale bar is 12 μm. The arrows indicate crystallographic directions *a* and *b*. (d and e) Polar plots of *a*_1_ in orange and *a*_2_ in blue, respectively, as extracted from fits to *A*(θ) for sample Co-1. In panel e, the red curve is a fit to *b*_1_ sin ^2^θ + *b*_2_ cos ^2^θ. Angle θ is defined with respect to the crystallographic *b*-axis.

Reference (9) shows that the magnetostriction coefficient in MPS_3_ materials is anisotropic; therefore, investigating if the thermo-magnetostrictive driving force also depends on the crystal orientation is interesting. We study this by applying the aforementioned analysis to determine the *a*_1_ and *a*_2_ fit parameters for all rectangular CoPS_3_ resonators of sample Co-1. As derived in Supporting Information H, the parameters are expected to follow the angular dependence as
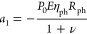
4
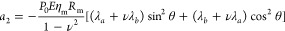
5where η_ph_ and η_m_ are the fractions of heat absorbed by the phononic and magnetic baths, respectively, ν is the Poisson ratio, *E* is Young’s modulus, λ_*a*_ and λ*_b_* are the magnetostriction coefficients along the *a*- and *b*-axes, respectively, and θ is defined with respect to the *b* direction. Thus, from eq 4, we expect *a*_1_ to be isotropic, which agrees with the polar plot in Figure 4d showing the extracted *a*_1_ parameters from the fit to angle-resolved data measured on the rectangular CoPS_3_ resonators. Similarly, *a*_2_(θ) reproduces the anisotropic function in eq 5, as shown via a fit (red line in Figure 4e) to a function of the form *b*_1_ sin ^2^θ + *b*_2_ cos ^2^θ. The qualitative agreement with eq 5 provides additional evidence that the driving force is of magnetostrictive origin and is consistent with earlier observations of the anisotropy in the magnetostriction coefficient of CoPS_3_.^9^

We present evidence that resonators of antiferromagnetic 2D materials can be driven via a thermo-magnetostrictive effect. The effect was shown in FePS_3_, CoPS_3_, and NiPS_3_ (Figure S11) and can lead to strong enhancement of the thermomechanical force below the Neél temperature, because its magnitude scales with the temperature derivative of the square of the staggered magnetization, . Despite its magnetic nature, the effect does not require application of external magnetic fields and is also effective in antiferromagnetic materials, because the magnetization is varied by optothermal modulation.

As a consequence of the anisotropy in the magnetostriction coefficient, we observe a strong crystal orientation dependence in the amplitude of the thermo-magnetostrictive driving force. In addition to providing a route toward driving magnetic nanomechanical resonators, the observed high-frequency magnetostrictive effects can provide further insights into the interplay among motion, thermodynamics, magnetic order, and mechanical strain in the 2D limit.

## Data Availability

All data supporting the findings of this article and its Supporting Information will be made available upon request to the authors.

## Supporting Information Available

Experimental methods and measured sample (A), sources of delay between membrane displacement and optical actuation (B), specific heat from resonance frequency (C), thermal conductivity from the thermal time constant (D), optothermal actuation of magnetic membranes (E), order parameter from the resonance frequency (F), additional data for MPS_3_ resonators (G), and angle-resolved membrane displacement (H) (PDF)
