# Supplementary material for: Thermo-Magnetostrictive Effect for Driving Antiferromagnetic Two-Dimensional Material Resonators
Source: Nano Lett. 2023 Jul 19;23(15):6973–8. doi: 10.1021/acs.nanolett.3c01610 (PMC10416344; doi:10.1021/acs.nanolett.3c01610)
Supplement: Supplementary file 1 — nl3c01610_si_001.pdf [file nl3c01610_si_001.pdf]

Thermo-magnetostrictive effect for driving antiferromagnetic 2D material resonators Supplementary Information

Gabriele Baglioni,[*,†] Makars Šiškins,[†] Maurits Houmes,[†] Martin Lee,[†] Dong Hoon Shin,[†] Samuel Mañas-Valero,[†,‡] Eugenio Coronado,[‡] Yaroslav M. Blanter,[†] Herre S.J. van der Zant,[†] and Peter G. Steeneken[*,†,¶]

†*Kavli Institute of Nanoscience, Delft University of Technology, The Netherlands, Lorentzweg 1, 2628 CJ Delft, The Netherlands*

‡*Instituto de Ciencia Molecular (ICMol), Universitat de Valencia, Catedrático José Beltrán 2, 46980 Paterna, Spain*

¶*Department of Precision and Microsystems Engineering, Delft University of Technology, The Netherlands, Mekelweg 2, 2628 CD Delft, The Netherlands*

E-mail: G.Baglioni@tudelft.nl; P.G.Steeneken@tudelft.nl

A. Experimental methods and measured samples

Sample fabrication

MPS_3 flakes are stamped onto a substrate of 285 nm thermal SiO_2, grown on highly doped (Si^{++}) silicon. Cavities are defined via e-beam lithography using AR-P 6200 resist. After development, the exposed SiO_2 areas are fully etched via reactive ion etching. The AR-P 6200 resist is stripped in PRS-3000 and the sample is cleaned in an O_2 plasma before

stamping. The exfoliation and transfer of multi-layer MPS_3 flakes is done using a PDMS transfer method. First, MPS_3 crystals are exfoliated onto the PDMS through scotch tape. Selected flakes are then transferred either on the circle-shaped or on the star-shaped cavities in the SiO_2/Si substrate.

Laser interferometry

The samples are mounted in a 4K cryostat (Montana Instruments Cryostation s50) with optical access. An intensity-modulated blue laser (405nm) causes the membrane to heat up, resulting in its deflection due to thermal expansion. The motion of the membrane is detected via cavity optomechanics using a red He-Ne laser (632 nm). The suspended membrane acts as a moving mirror and the bottom of the cavity as a fixed mirror in a low-finesse Fabry-Perot cavity. The reflected red laser light from the cavity is redirected to a photodetector, which is connected to a vector network analyzer (VNA) in order to measure transmission between the blue laser modulation and the signal on the photodetector in a homodyne detection scheme. The sample is mounted on a heater stage used to control the sample local temperature inside the cryostat with 10 mK precision. Measurements are performed at incident laser power of $< 10\mu$W for the red laser and $< 1\mu$W for the blue laser. Figure S1 shows a schematic of the experimental setup described.

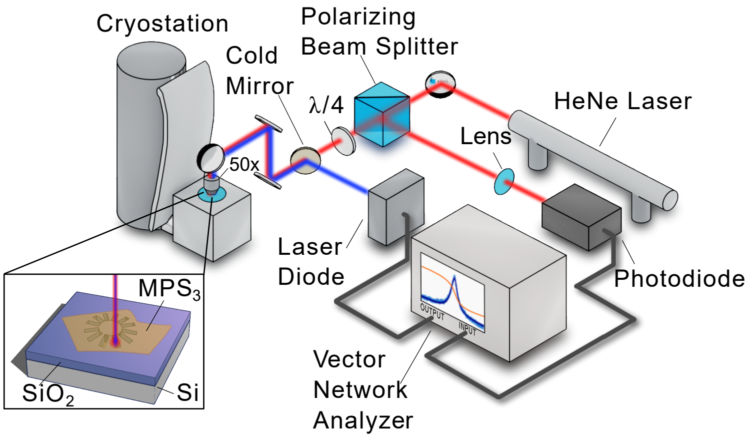

Figure S1: **Experimental setup.** Schematic of the interferometry setup.

Crystal growth

Crystal growth of MPS_3 (M = Ni, Fe, Co) was performed following a solid-state reaction inside a sealed evacuated quartz tube (pressure $\sim 5 \times 10^{-5}$ mbar). I_2 was used as a transport agent to obtain large crystals. A three zone furnace was used, where a tube with the material was placed in the leftmost zone. The leftmost side was then heated up to 700 °C in 3 hours so that a temperature gradient of 700 °C/650 °C/675 °C was established. The other two zones were heated up in 24 hours from room temperature to 650 °C and kept at that temperature for one day. The temperature was kept constant for 28 days and cooled down naturally. With this process crystals with a length up to several centimeters are obtained. Detailed description of the crystal growth and characterization can be found in earlier work.[1]

Measured samples

Table S1 shows an overview of the measured samples of MPS_3 resonators, along with their dimensions and flake thickness as measured via atomic force microscopy.

Table S1: **Measured samples.** Overview of the MPS_3 samples studied. Reported are the material, the resonator's geometry, the resonator's dimensions (radius, r, for circular drums and width, w, and lenght, l, for rectangular membranes in star cavity) and thickness, t, as determined by atomic force microscopy (AFM).

Sample	Material	Resonator geometry	Dimensions	t (nm)
Fe-1	$FePS_3$	Circular drum	$r = 6\,\mu$m	16
Fe-2	$FePS_3$	Circular drum	$r = 4\,\mu$m	21
Fe-3	$FePS_3$	Star cavity array	$w = 2\,\mu$m, $l = 16\,\mu$m	40
Co-1	$CoPS_3$	Star cavity array	$w = 2\,\mu$m, $l = 16\,\mu$m	30
Ni-1	$NiPS_3$	Circular drum	$r = 4\,\mu$m	16
Ni-2	$NiPS_3$	Circular drum	$r = 4\,\mu$m	20

B. Sources of delay between membrane displacement and optical actuation

In the following, we discuss the possible sources for time delay between the modulation of the actuating blue laser and the membrane displacement. As argued in Ref. [2], the heating power caused by the modulated intensity can be treated as instantaneous as photoexcited carriers lose their energy to phonons on time scales of a few picoseconds. Then, the heating power will increase the membrane temperature in a time scale determined by the membrane thermal time constant τ. Thus, the resulting force acting on the membrane will also be delayed by τ with respect to the optical actuation. Additional mechanical delays are also neglected, since for frequencies far below the membrane's resonance frequency, the membrane motion will be in phase with the actuating force.

Other sources of delay, intrinsic to the measurement setup are taken into account by pointing directly the blue laser to the photodiode to obtain a calibration curve c_ω for the frequency response f_ω. The corrected frequency response is given by

$$f_{\text{corr},\,\omega} = \frac{f_\omega}{c_\omega}\,. \tag{S1}$$

Thus, we conclude that the dominant delay source is the thermal time constant of the system. Additional delays between the change in temperature and change in magnetization causing the magnetostriction force are neglected as the data is well represented by a single time constant model.

C. Specific heat from resonance frequency

Following the model from Ref. [1], we derived the specific heat c_v from the temperature derivative of the resonance frequency $f_0 = \omega_0/2\pi$ as

$$c_v = 3\left(\alpha_{\mathrm{Si}} - \frac{1}{\xi^2}\frac{df_0^2}{dT}\right)\frac{EV_M}{3\gamma(1-2\nu)}, \tag{S2}$$

where α_{Si} is the thermal expansion coefficient of Si, E is the Young's modulus, ν is the Poisson ratio, $\gamma \approx \frac{3}{2}\frac{1+\nu}{2-3\nu}$ is the Grüneisen parameter, V_M is the molar volume and $\xi = \frac{2.4048}{\pi d}\sqrt{\frac{E}{\rho(1-\nu)}}$, with ρ the density of the material.

The c_v of $FePS_3$ shown in Fig. 3a of the main text is computed via Eq. S2 from the resonance frequency data shown in Fig. S2, with E = 103 GPa, ν = 0.304, ρ = 3375 kg m^{-3}.

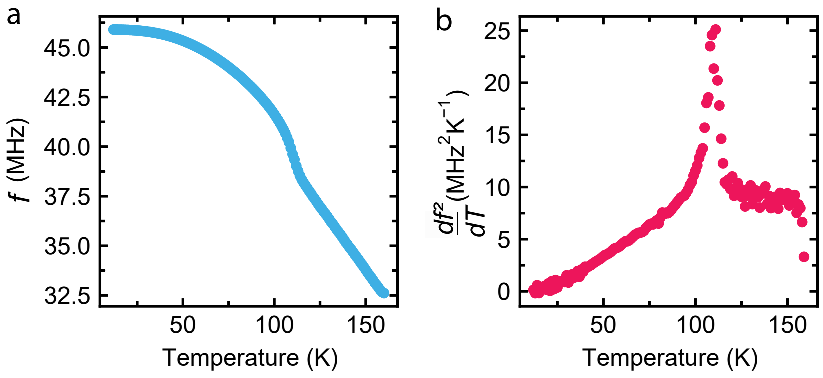

Figure S2: **Data on $\omega_0/2\pi$ for sample Fe-1 (a).** Temperature dependent resonance frequency, $\omega_0(T)/2\pi$ of sample Fe-1.**b** Derivative with respect to temperature of $\omega_0/2\pi$ from (a).

D. Thermal conductivity from thermal time constant

Instead of using the literature value of κ to determine τ as done in the main text, we could also use the measured value of τ to estimate the membrane's thermal conductivity κ. Such an analysis would be of particular interest for materials whose bulk values of κ are not

reported, like for $CoPS_3$, or to study thermal conductivity in the thin limit. However, we note that prior knowledge of the material's mechanical properties, such as Young's modulus and Poisson ratio, are needed to estimate c_v from $\frac{d\omega_0^2}{dT}$. We also note that more clarification is needed to account for the relatively high value of the parameter $\mu^2 = 10$ compared to previous work,[3] where a value of $\mu^2 = 5$ was found close to the theoretical value for a circular membrane of $\mu^2 = 5.783$.

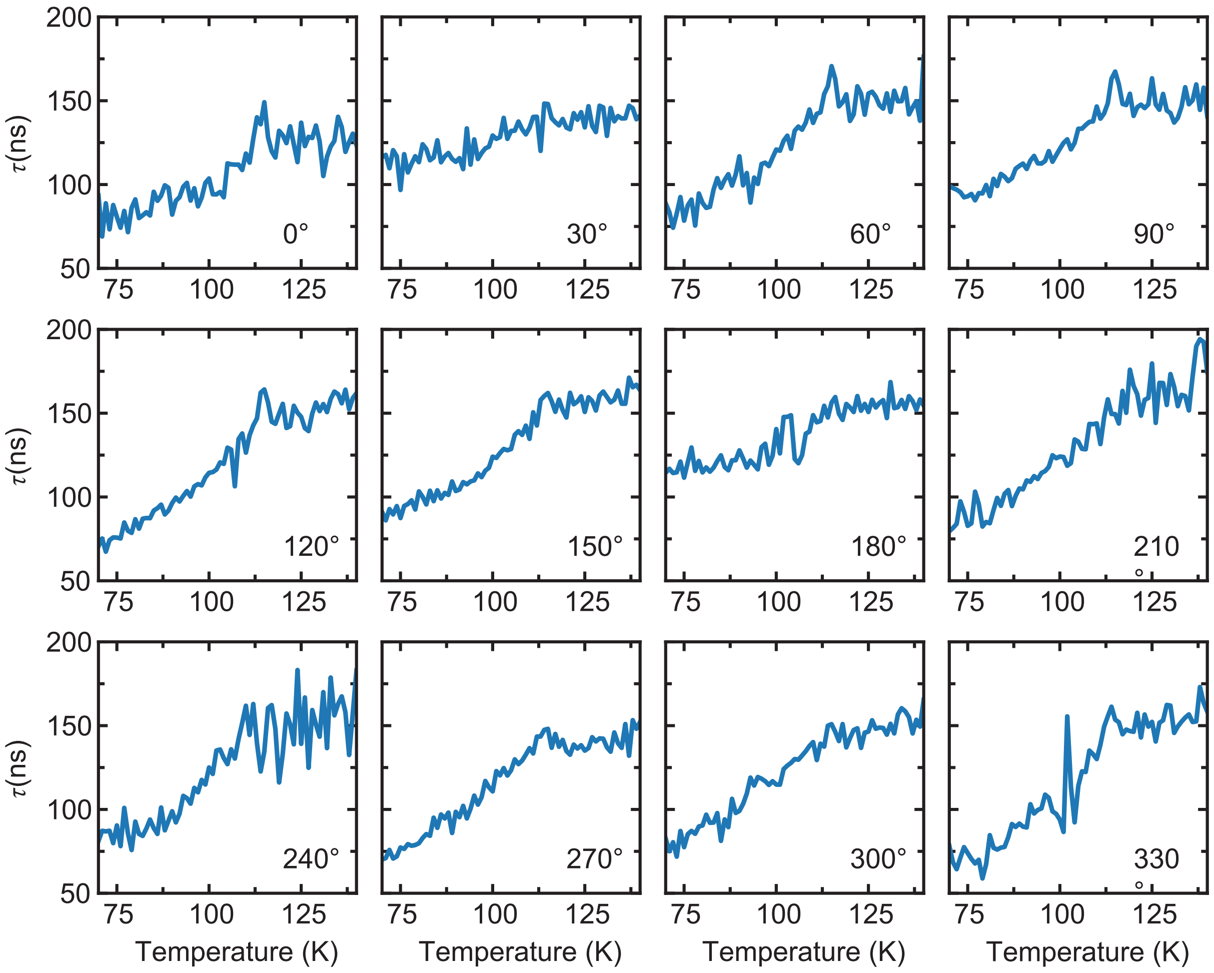

Figure S3: **Angle-resolved thermal time constant in $CoPS_3$ resonators.** Thermal time constant rectangular cavities at different orientation with respect to the crystallographic axis of the Co-1 sample.

Despite the fact that we do not yet obtain a full quantitative agreement between the model and theory, here we present an estimate of the angle-resolved thermal conductivity,

k, of $CoPS_3$ from the thermal time constants, τ measured on the rectangular resonators of sample Co-1 (these are shown in Fig. S3). We calculate k as[4]

$$k = \frac{w^2 \rho c_v}{\pi^2 \tau}, \tag{S3}$$

where $w = 2\mu$m is the width of the resonator, $\rho = 3257$ kg m^{-3}, and c_v is the bulk specific heat taken from Ref. [5]. The resulting k for all resonators is shown in Fig. S4.

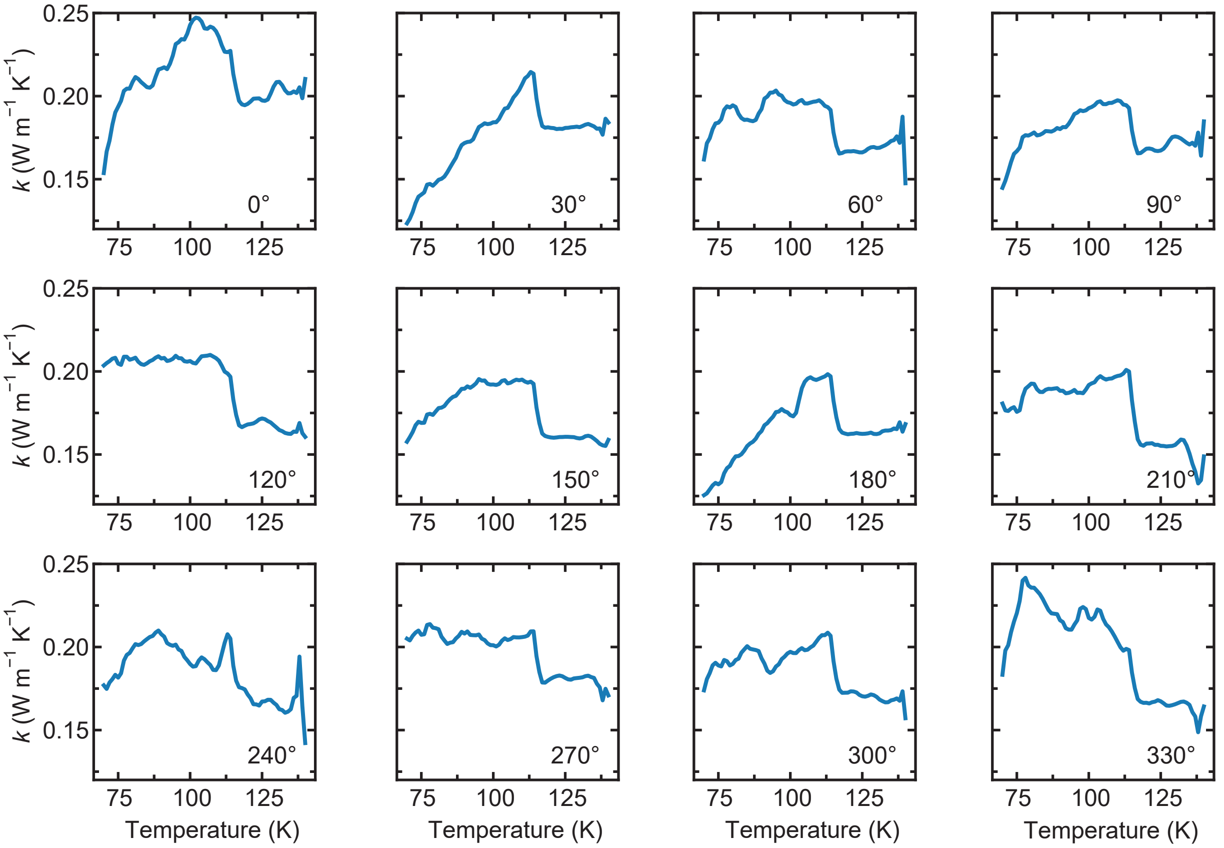

Figure S4: **Angle-resolved thermal conductivity in $CoPS_3$ resonators.** Estimated thermal conductivity of rectangular cavities at different orientation with respect to the crystallographic axis of the Co-1 sample. The thermal conductivity is calculated from τ data shown in Fig. S3 using Eq. S3

E. Optothermal actuation of magnetic membranes

Here we derive Eq. 2 of the main text. Let us consider the temperature dynamics of the system under optothermal actuation from the power-modulated blue laser, $P_\omega = P_0 e^{i\omega t}$. We start by treating separately the temperature dynamics of the magnetic and phononic systems as $T_{\omega,\mathrm{ph}}$ and $T_{\omega,\mathrm{m}}$, as illustrated in Fig. S5. The fraction of power absorbed from P_ω by each system is determined by the phononic absorption fraction, η_{ph}, and magnetic absorption fraction, η_{m}. The temperature dynamics of the two systems is expressed as a simple RC system:

$$C_{\mathrm{ph}} \frac{dT_{\omega,\mathrm{ph}}}{dt} + \frac{1}{R_{\mathrm{ph}}} T_{\omega,\mathrm{ph}} = P_0 e^{i\omega t}\,, \tag{S4}$$

$$C_{\mathrm{m}} \frac{dT_{\omega,\mathrm{m}}}{dt} + \frac{1}{R_{\mathrm{m}}} T_{\omega,\mathrm{m}} = P_0 e^{i\omega t}\,, \tag{S5}$$

where C and R are the system's thermal capacitance and thermal resistance.

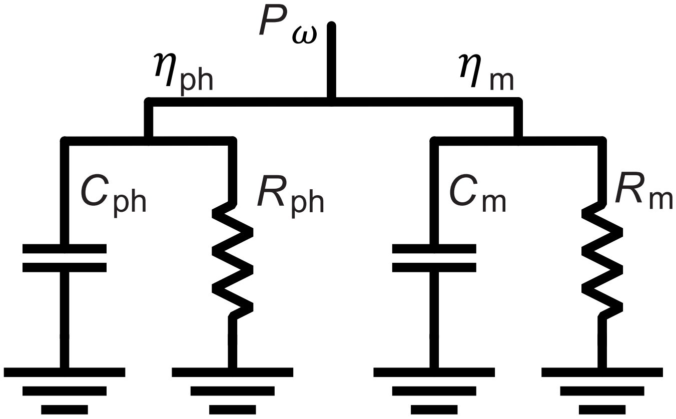

Figure S5: **Equivalent circuit illustration of the phononic and magnetic thermal system.** The input power, P_ω, from the blue laser is absorbed with different fractions, η_{ph} and η_{m}, by the phononic and magnetic systems. The corresponding temperature dynamics is determined by the system thermal resistance, R, and capacitance, C.

Thus, the temperature dynamics of the phononic and magnetic systems is given by:

$$T_{\omega,\mathrm{ph}} = \frac{\eta_{\mathrm{ph}} R_{\mathrm{ph}}}{1 + i\omega\tau_{\mathrm{ph}}} P_0 e^{i\omega t}\,, \tag{S6}$$

$$T_{\omega,\mathrm{m}} = \frac{\eta_{\mathrm{m}} R_{\mathrm{m}}}{1 + i\omega\tau_{\mathrm{m}}} P_0 e^{i\omega t}\,, \tag{S7}$$

where $\tau_{\mathrm{ph}} = R_{\mathrm{ph}} C_{\mathrm{ph}}$ and $\tau_{\mathrm{m}} = R_{\mathrm{m}} C_{\mathrm{m}}$ are the time constants of the two systems.

Below T_{N}, the temperature modulation T_ω determines changes in the thermal expansion force, $F_{\mathrm{th}} = \alpha T$, where α is the thermal expansion coefficient and the magnetostriction force, $F_{\mathrm{ms}} = \lambda L^2(T)$, where λ is a magnetostriction coefficient and L the order parameter. When $\omega \ll \omega_0$, the membrane behaves simply as a spring with stiffness k, such that its motion is given by $z_\omega = F_\omega / k$. Thus, given the force modulation resulting from T_ω we get

$$z_\omega = \frac{1}{k} \left(\alpha T_{\omega,\mathrm{ph}} + \lambda \frac{dL^2}{dT_{\omega,\mathrm{m}}} T_{\omega,\mathrm{m}} \right)\,. \tag{S8}$$

Substituting Eqs. S6 and S7 in S8 yields:

$$z_\omega = \frac{1}{k} \left(\alpha \frac{\eta_{\mathrm{ph}} R_{\mathrm{ph}} P_0}{1 + i\omega\tau_{\mathrm{ph}}} + \lambda \frac{dL^2}{dT_{\omega,\mathrm{m}}} \frac{\eta_{\mathrm{m}} R_{\mathrm{m}} P_0}{1 + i\omega\tau_{\mathrm{m}}} \right)\,. \tag{S9}$$

Since the measured data is well represented by a single time constant model, we assume that the temperature dynamics of the phononic and magnetic systems are the same, $\tau_{\mathrm{ph}} = \tau_{\mathrm{m}}$. Thus, Eq. S9 is Eq. 2 of the main text.

F. Order parameter from resonance frequency

As derived in [6], the general equation of the resonance frequency, f_θ, of a magnetic rectangular membrane oriented at an angle θ with respect to the crystalline axes is given by:

$$f_\theta(T) = \frac{1}{2} \sqrt{\frac{E}{\rho w^2 (1 - \nu^2)} \left[(\sin^2\theta + \nu \cos^2\theta)(\bar{\epsilon}_a + \epsilon_{\lambda,a}) + (\cos^2\theta + \nu \sin^2\theta)(\bar{\epsilon}_b + \epsilon_{\lambda,b}) \right]}\,, \tag{S10}$$

where $\bar{\epsilon}_{a,b} = \epsilon_{0,a,b} + \epsilon_{\alpha,a,b}$, with $\epsilon_{0,a,b}$ the residual fabrication strain and $\epsilon_{\alpha,a,b}$ the thermal expansion strain and $\epsilon_{\lambda,a,b} = \lambda_{a,b} L^2$ are the magnetostrictive strain along the a-axis and b-axis. Under the assumption that the only anisotropic temperature-dependent contribution

to the total strain comes from magnetostriction ($\epsilon_{\alpha,a} = \epsilon_{\alpha,b}$), we have

$$\tilde{f}_a^2 - \tilde{f}_b^2 = \frac{E}{4\rho w^2(1+\nu)}(\lambda_a - \lambda_b)L^2\,, \tag{S11}$$

where $\tilde{f}^2 = f^2(T) - f^2(T_0)$, with T_0 a reference temperature, is the pretension corrected resonance frequency.

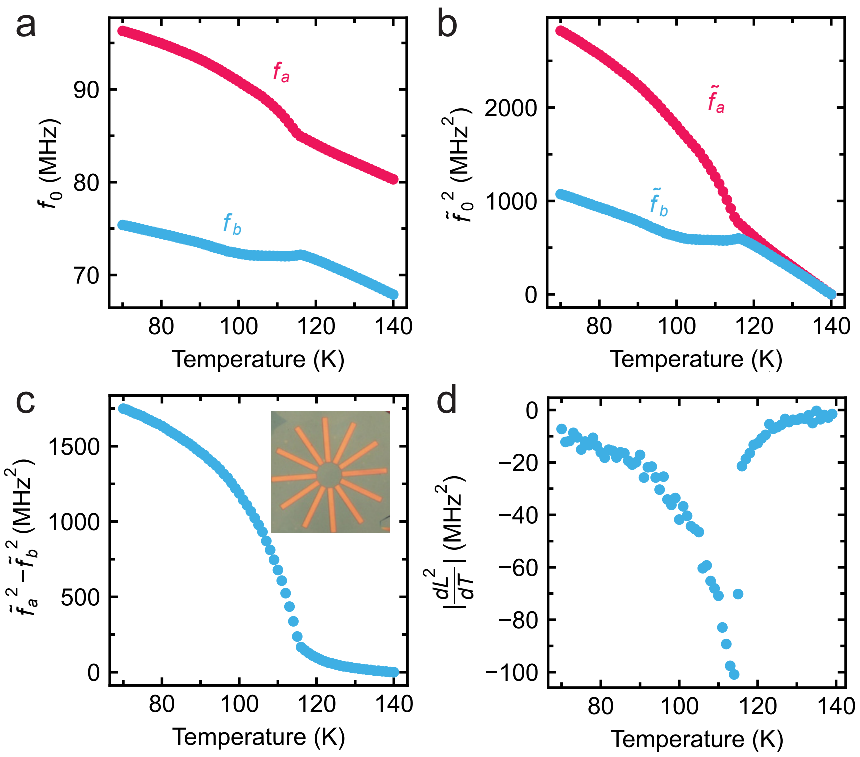

Figure S6: **Magnetostriction force from resonance frequency of $CoPS_3$.** Measurements and analysis performed on sample Co-1. **(a)** Bare resonance frequency, $f_\theta(T)$, measured on rectangular membranes oriented along the main crystallographic axes. **(b)** Pretension corrected resonance frequency, $\tilde{f}^2 = f^2(T) - f^2(T_0)$, with $T_0 = 140$K. **(c)** Difference between corrected resonance frequencies, $\tilde{f}_a^2 - \tilde{f}_b^2$, proportional to the magnetic order parameter, L^2. The inset shows the $CoPS_3$ sample Co-1. **(d)** Magnetostriction force.

We use Eq. S11 to extract magnetostriction force, $F_{\rm ms} \propto \dfrac{dL^2}{dT}$, used to model the thermal peak amplitude, $A(T)$, in the main text. Figure S6 shows the steps of this analysis for the Co-1 sample, also shown in the inset of Fig. S6c. The bare resonance frequencies f_a and f_b measured on rectangular cavities oriented along a and b crystallographic axis are shown

in Fig. S6a. First, we compute the pretension corrected resonance frequency, shown in Fig. S6b, with $T_0 = 140$ K, to remove the contribution of ϵ_0 in Eq. S10. Then, following Eq. S11, the difference between $\tilde{f}^2$ for membranes oriented along different axes is proportional to L^2 (Fig. S6c). Finally, the temperature dependance of the magnetostriction force F_{ms} is found by taking the temperature derivative of $\tilde{f}_a^2 - \tilde{f}_b^2$, as shown in Fig. S6d.

Figure S7 shows how the resulting magnetostriction force is combined with the Debye model of specific heat to fit the experimental data of the thermal peak amplitude of a rectangular membrane of sample Co-1. The results of this analysis performed for all rectangular membranes of sample Co-1 are shown in Fig. S9.

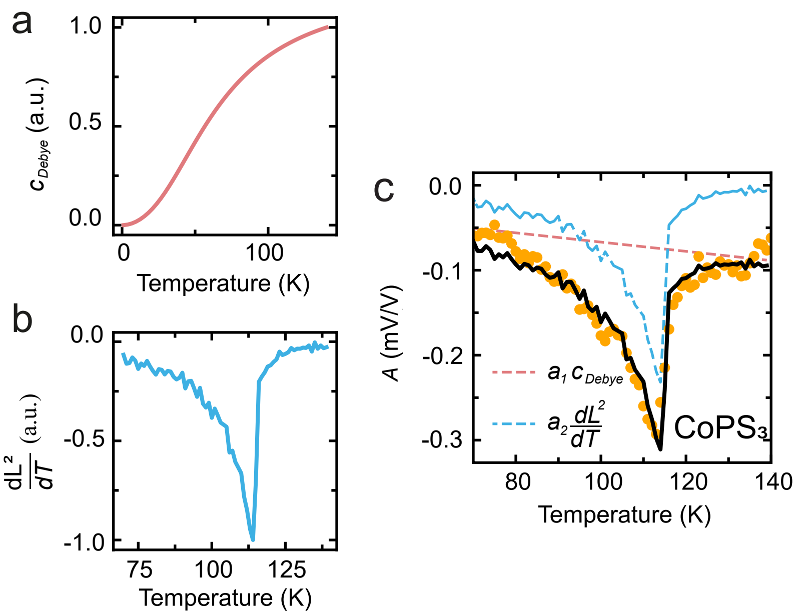

Figure S7: **Model for thermal peak amplitude.** Thermal peak amplitude fit on a rectangular resonator of sample Co-1. **(a)** Debye model of the phononic specific heat with $\Theta_D = 262$ K. **(b)** Magnetostriction force extracted from antiferromagnetic order parameter, L. **(c)** Thermal peak amplitude data (orange dots) compared to model (black) and contributions from phononic specific heat (light red) and magnetostriction force (light blue).

On Fig. S8, we show the same analysis done in Fig. S6 for the $FePS_3$ sample Fe-3. We use the resulting temperature dependance of the magnetostriction force from Fig. S8d to model the thermal peak amplitude data of the $FePS_3$ sample Fe-1 in Fig.4b of the main text and of the sample Fe-2 in Fig. S10 of Supplementary Information.

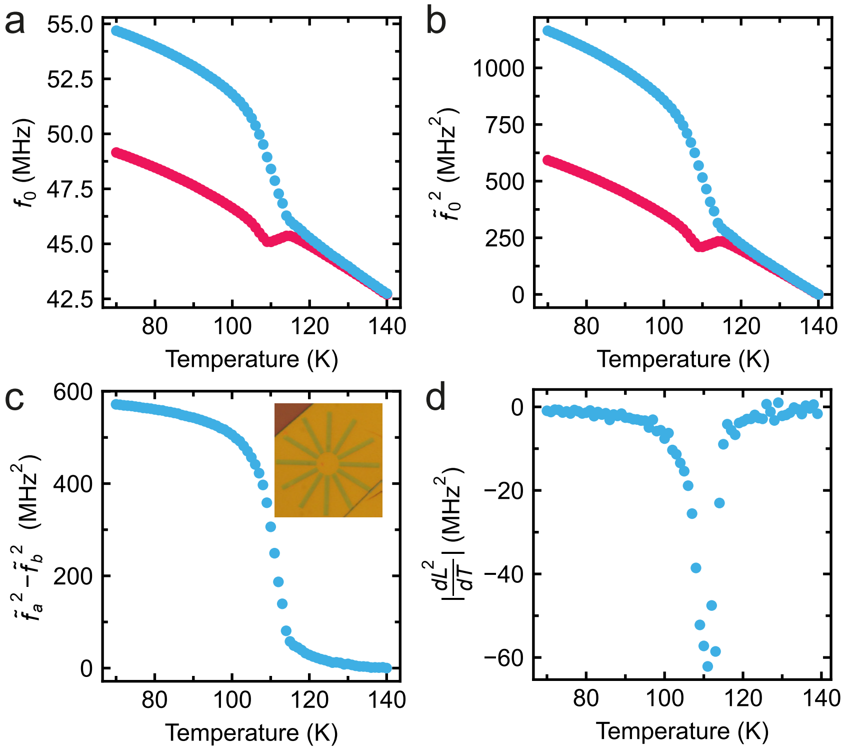

Figure S8: **Magnetostriction force from resonance frequency of $FePS_3$.** Measurements and analysis performed on sample Fe-3 **(a)** Bare resonance frequency, $f_\theta(T)$, measured on rectangular membranes oriented along the main crystallographic axes. **(b)** Pretension corrected resonance frequency, $\tilde{f}^2 = f^2(T) - f^2(T_0)$, with $T_0 = 140$K. **(c)** Difference between corrected resonance frequencies, $\tilde{f}_a^2 - \tilde{f}_b^2$, proportional to the magnetic order parameter, L^2. The inset shows the $FePS_3$ sample Fe-3. **(d)** Magnetostriction force.

G. Additional data on MPS_3 resonators

Figure S9 shows the result of the analysis done in Supplementary Information F for the thermal peak amplitude measured on all rectangular cavities of sample Co-1. The resulting fit parameters $a_1(\theta)$ and $a_2(\theta)$ are listed in Table S2 plotted in Fig.4d-e of the main text.

Figures S10-S11 show additional data on $FePS_3$ (sample Fe-2) and $NiPS_3$ (sample Ni-1,2) resonators. Interestingly, in $NiPS_3$ no peak is observed at the phase transition on the thermal time constant data. The large enhancement of the thermal peak amplitude, A is still observed. Further analysis and theoretical work on thermal peak amplitude data might allow the extraction of the order parameter, L, of $NiPS_3$ from the measured A instead.

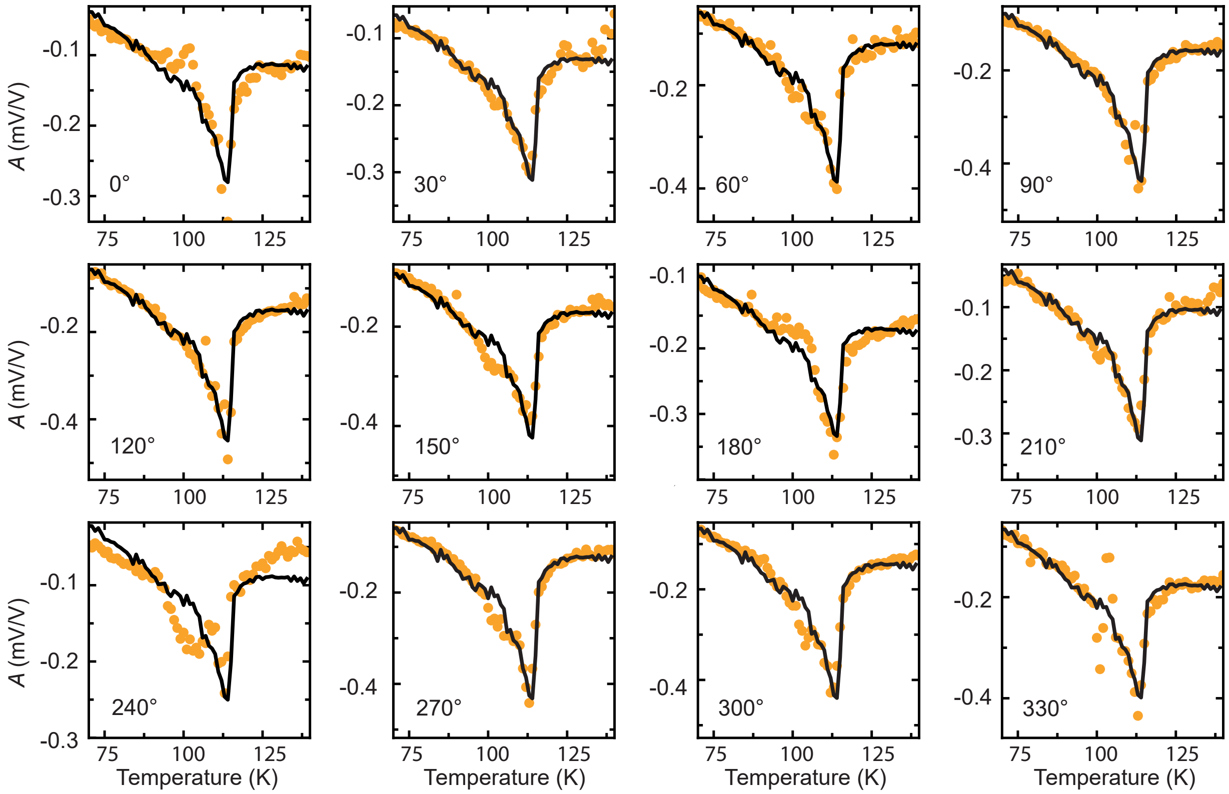

Figure S9: **Angle-resolved thermal peak amplitude in $CoPS_3$ resonators.** Thermal peak amplitude data (orange dots) compared to model (black) for rectangular cavities at different orientation with respect to the crystallographic axis of the Co-1 sample.

Table S2: **Angle-resolved fit parameters a_1 and a_2** Fit parameters a_1 and a_2 for the plots in Fig. S9.

Angle	a_1	a_2
0°	-0.29	0.19
30°	-0.29	0.20
60°	-0.29	0.29
90°	-0.31	0.31
120°	-0.31	0.33
150°	-0.31	0.28
180°	-0.29	0.18
210°	-0.29	0.23
240°	-0.29	0.18
270°	-0.30	0.33
300°	-0.31	0.32
330°	-0.29	0.25

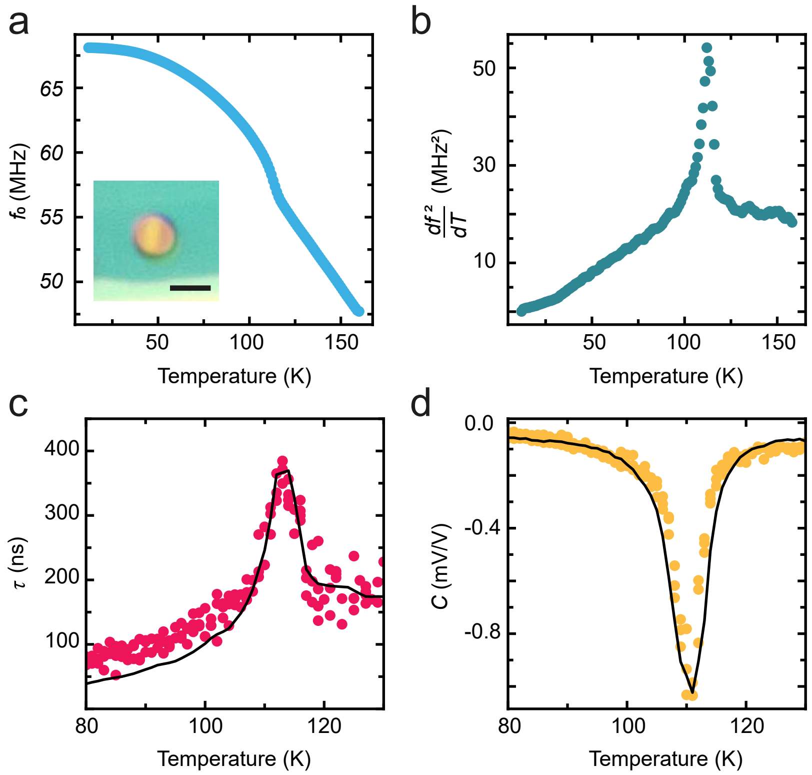

Figure S10: **Additional data on $FePS_3$ resonators.** Measurements data and analysis performed on sample Fe-2. **(a)** Temperature dependent resonance frequency, $f_0(T)$. The inset shows the $FePS_3$ sample Fe-2. Scale bar is 4μm. **b** Derivative with respect to temperature of f_0 from (a). **(c)** Comparison between measured τ (red dots) and model (black line). The model is calculated from Eq. 1 from the main text with c_v from df^2/dT in (b), κ from bulk[7] and $\mu^2 = 10$. **(d)** Thermal peak amplitude data (orange dots) compared to model (black).

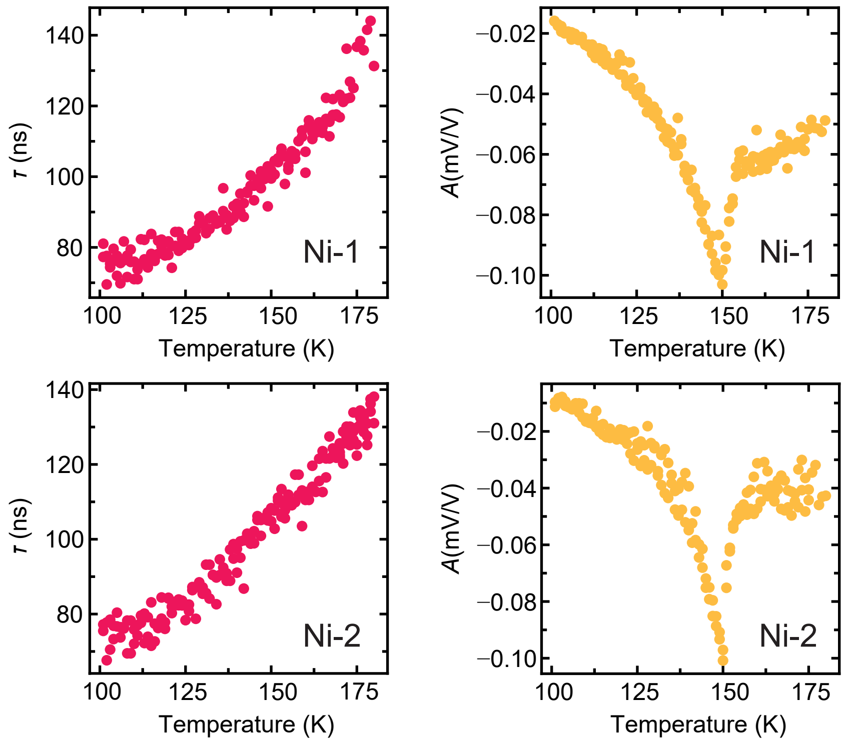

Figure S11: **Additional data on $NiPS_3$ resonators.** **(Left)** Thermal time constant of the $NiPS_3$ resonators. **(Right)** Thermal peak amplitude of the $NiPS_3$ resonators.

H. Angle-resolved membrane displacement

Here we derive the expression of $a_1(\theta)$ and $a_2(\theta)$ used in the main text. Given the stress σ on a rectangular membrane oriented at an angle θ with respect to the crystalline axis:

$$\sigma_x = \sigma_a \cos^2\theta + \sigma_b \sin^2\theta\,, \tag{S12}$$

and

$$\sigma_y = \sigma_a \sin^2\theta + \sigma_b \cos^2\theta\,, \tag{S13}$$

If such stress is time dependent, it will actuate the membrane as $z(t) = \gamma_w\sigma_x + \gamma_l\sigma_y$, where γ is an effective expansion coefficient. From the constitutive equations of the materials, we

can write

$$c_1 = E(\epsilon_{0,a} + \epsilon_{\alpha,a} + \epsilon_{\lambda,a}) = E\left(\epsilon_{0,a} - \int_{T_0}^{T_1} \alpha_a(T)dT - \lambda_a L^2(T_1)\right) = \sigma_a(T_1) - \nu\sigma_b(T_1)\,, \tag{S14}$$

$$c_2 = E(\epsilon_{0,b} + \epsilon_{\alpha,b} + \epsilon_{\lambda,b}) = E\left(\epsilon_{0,b} - \int_{T_0}^{T_1} \alpha_b(T)dT - \lambda_b L^2(T_1)\right) = \sigma_b(T_1) - \nu\sigma_a(T_1)\,, \tag{S15}$$

where ϵ_0 is residual fabrication strain at $T = T_0$, α is the thermal expansion coefficient, λ the magnetostriction coefficient and E is the Young's modulus, which is assumed to be isotropic. We can thus write

$$\sigma_a = c_1 + \nu\sigma_b\,, \tag{S16}$$

$$\sigma_b = c_2 + \nu\sigma_a\,, \tag{S17}$$

which can be combined in the following expressions for σ_a and σ_b:

$$\sigma_a = \frac{c_1 + \nu c_2}{1 - \nu^2}\,, \tag{S18}$$

$$\sigma_b = \frac{c_2 + \nu c_1}{1 - \nu^2}\,. \tag{S19}$$

We consider separately the temperature dynamics of the magnetic and phononic systems as $T_{\omega,\mathrm{m}}$ and $T_{\omega,\mathrm{ph}}$ as in Eqs. S7 and S6. The membrane displacement can thus be expressed as

$$z_\theta(t) = \gamma_w\sigma_x(T_{\omega,\mathrm{ph}}(t), T_{\omega,\mathrm{m}}(t)) + \gamma_l\sigma_y(T_{\omega,\mathrm{ph}}(t), T_{\omega,\mathrm{m}}(t)) \tag{S20}$$

$$\approx \gamma_w\left[\frac{\partial\sigma_x}{\partial T_{\omega,\mathrm{ph}}}T_{\omega,\mathrm{ph}}(t) + \frac{\partial\sigma_x}{\partial T_{\omega,\mathrm{m}}}T_{\omega,\mathrm{m}}(t)\right] + \gamma_l\left[\frac{\partial\sigma_y}{\partial T_{\omega,\mathrm{ph}}}T_{\omega,\mathrm{ph}}(t) + \frac{\partial\sigma_y}{\partial T_{\omega,\mathrm{m}}}T_{\omega,\mathrm{m}}(t)\right]\,. \tag{S21}$$

Assuming that thermal expansion is isotropic ($\alpha_x = \alpha_y$) and that it only depends on $T_{\omega,\mathrm{ph}}$ and that magnetostriction only depends on $T_{\omega,\mathrm{m}}$ we have

$$\frac{\partial c_1}{\partial T_{\omega,\mathrm{ph}}} = -E\alpha(T_{\omega,\mathrm{ph}}), \qquad \frac{\partial c_1}{\partial T_{\omega,\mathrm{m}}} = -E\lambda_a \frac{\partial L^2}{\partial T_{\omega,\mathrm{m}}}(T_{\omega,\mathrm{m}}), \tag{S22}$$

$$\frac{\partial c_2}{\partial T_{\omega,\mathrm{ph}}} = -E\alpha(T_{\omega,\mathrm{ph}}), \qquad \frac{\partial c_2}{\partial T_{\omega,\mathrm{m}}} = -E\lambda_b \frac{\partial L^2}{\partial T_{\omega,\mathrm{m}}}(T_{\omega,\mathrm{m}}), \tag{S23}$$

Thus, combining Eqs. S22, S23 and S24 we get:

$$z_\theta(t) = -\frac{P_0 E}{(1-\nu^2)}\left[\frac{(\gamma_w+\gamma_l)\eta_{\mathrm{ph}}R_{\mathrm{ph}}(1+\nu)}{1+i\omega\tau_{\mathrm{ph}}}\alpha + \right. \tag{S24}$$

$$+ \frac{\eta_{\mathrm{m}}R_{\mathrm{m}}}{1+i\omega\tau_{\mathrm{m}}}\Big((\gamma_w(\lambda_a+\nu\lambda_b)+\gamma_l(\lambda_b+\nu\lambda_a))\cos^2\theta + \tag{S25}$$

$$\left. + (\gamma_w(\lambda_b+\nu\lambda_a)+\gamma_l(\lambda_a+\nu\lambda_b))\sin^2\theta\Big)\right]. \tag{S26}$$

For high aspect-ratio rectangular membranes ($w \ll l$), it is possible to neglect the force along the long axis $\gamma_w \ll \gamma_l$ such that

$$z_\theta(t) = -\frac{P_0\gamma_l E}{(1-\nu^2)}\left[\frac{\eta_{\mathrm{ph}}R_{\mathrm{ph}}(1+\nu)}{1+i\omega\tau_{\mathrm{ph}}}\alpha + \frac{\eta_{\mathrm{m}}R_{\mathrm{m}}}{1+i\omega\tau_{\mathrm{m}}}\Big((\lambda_b+\nu\lambda_a)\cos^2\theta + (\lambda_a+\nu\lambda_b)\sin^2\theta\Big)\right], \tag{S27}$$

from which the Eq. 4 and 5 for $a_1(\theta)$ and $a_2(\theta)$ of the main text are extracted.

Supplementary References

1. Šiškins, M.; Lee, M.; Mañas-Valero, S.; Coronado, E.; Blanter, Y. M.; van der Zant, H. S. J.; Steeneken, P. G. Magnetic and electronic phase transitions probed by nanomechanical resonators. *Nat. Commun.* **2020**, *11*, 2698.

2. Dolleman, R. J.; Houri, S.; Davidovikj, D.; Cartamil-Bueno, S. J.; Blanter, Y. M.; van der Zant, H. S. J.; Steeneken, P. G. Optomechanics for thermal characterization of suspended graphene. *Phys. Rev. B* **2017**, *96*, 165421.

3. Dolleman, R. J.; Lloyd, D.; Lee, M.; Bunch, J. S.; van der Zant, H. S. J.; Steeneken, P. G. Transient thermal characterization of suspended monolayer MoS_2. *Phys. Rev. Materials* **2018**, *2*, 114008.

4. Lifshitz, R.; Roukes, M. L. Thermoelastic damping in micro- and nanomechanical systems. *Phys. Rev. B* **2000**, *61*, 5600–5609.

5. Liu, Q.; Wang, L.; Fu, Y.; Zhang, X.; Huang, L.; Su, H.; Lin, J.; Chen, X.; Yu, D.; Cui, X.; Mei, J.-W.; Dai, J.-F. Magnetic order in XY-type antiferromagnetic monolayer $CoPS_3$ revealed by Raman spectroscopy. *Phys. Rev. B* **2021**, *103*, 235411.

6. Houmes, M. J. A.; Baglioni, G.; Šiškins, M.; Lee, M.; Esteras, D. L.; Ruiz, A. M.; Mañas-Valero, S.; Boix-Constant, C.; Baldoví, J. J.; Coronado, E.; Blanter, Y. M.; Steeneken, P. G.; van der Zant, H. S. J. Magnetic order in 2D antiferromagnets revealed by spontaneous anisotropic magnetostriction. *arxiv [Preprint] https://doi.org/10.48550/arXiv.2303.11234* **submitted 20 Mar 2023, accessed 1 Apr 2023**,

7. Haglund, A. *Thermal Conductivity of* MXY_3 *Magnetic Layered Trichalcogenides*; Ph.D. thesis, University of Tennessee, Knoxville, 2019.
